# Probing SARS-CoV-2 Nsp8 Condensates with Neutron Scattering

**DOI:** 10.1063/4.0000984

**Published:** 2025-10-27

**Authors:** Sharique Khan, Wellinton Leite, Brighton Miller, Hugh O'Neill

**Affiliations:** 1 Oak Ridge National Laboratory, Oak Ridge, TN, USA; 2 University of Notre Dame, Notre Dame, IN, USA

## Abstract

The capacity of viral proteins to form biomolecular condensates through liquid-liquid phase separation (LLPS) has emerged as a key mechanism for organizing viral replication and transcription. In SARS-CoV-2, both the structural nucleocapsid (N) protein and nonstructural protein 8 (Nsp8) have been shown to undergo LLPS, yet the structural features and functional role of these higher order assemblies remain largely unexplored. Here, we show that Nsp8 robustly undergoes LLPS in vitro in the presence of a macromolecular crowder polyethylene glycol (PEG-8000), forming spherical droplets visualized by confocal microscopy. Fluorescence recovery after photobleaching (FRAP) confirms their liquid-like nature. Furthermore, Nsp7, a binding partner of Nsp8, co-localizes within these condensates, suggesting a regulatory role in condensate formation or function.

Preliminary small-angle neutron scattering (SANS) experiments using deuterated Nsp8 reveal a pronounced structure form factor peak around Q=0.0025 Å^-1^, the origin of which is still under investigation. The scattering profile suggests possible mesoscale organization within the system, but exact nature is still under investigation. Building up on this, we are designing follow-up SANS experiments using deuterated PEG and contrast-matching approaches to investigate the internal organization of the condensates with higher resolution.

We are also exploring the modulatory role of RNA, particularly homopolymeric U22, which has been shown to promote Nsp8 LLPS and may influence condensate architecture through multivalent interactions. Future work will examine how RNA alters the structural properties and stability of the condensates. In addition, we will assess whether Nsp8 retains its ATPase activity within the phase-separated state, providing critical insights into the functional relevance of LLPS during viral replication.

Together, these studies aim to elucidate the structural organization and biochemical behavior of Nsp8 within phase- separated condensates, advancing our understanding of viral replication mechanisms and identifying potential targets for therapeutic intervention.

